# Preventive Effects of Chitosan Coacervate Whey Protein on Body Composition and Immunometabolic Aspect in Obese Mice

**DOI:** 10.1155/2014/281097

**Published:** 2014-09-17

**Authors:** Gabriel Inácio de Morais Honorato de Souza, Aline Boveto Santamarina, Aline Alves de Santana, Fábio Santos Lira, Rachel de Laquila, Mayara Franzoi Moreno, Eliane Beraldi Ribeiro, Claudia Maria da Penha Oller do Nascimento, Bruno Rodrigues, Elisa Esposito, Lila Missae Oyama

**Affiliations:** ^1^Departamento de Fisiologia, Disciplina de Fisiologia da Nutrição, Universidade Federal de São Paulo, 04023-060 São Paulo, SP, Brazil; ^2^Departamento de Ciências Biológicas, Laboratório de Movimento Humano da Universidade São Judas Tadeu, 03166-000 São Paulo, SP, Brazil; ^3^Departamento de Educação Física da Universidade Estadual Paulista, Faculdade de Ciências e Tecnologia, 19060-900 Presidente Prudente, SP, Brazil; ^4^Departamento de Nutrição das Faculdades Integradas Coração de Jesus-FAINC, 09020-240 Santo André, SP, Brazil; ^5^Instituto de Ciências e Tecnologia da Universidade Federal de São Paulo, 04023-060 São José dos Campos, SP, Brazil

## Abstract

Functional foods containing bioactive compounds of whey may play an important role in prevention and treatment of obesity. The aim of this study was to investigate the prospects of the biotechnological process of coacervation of whey proteins (CWP) in chitosan and test its antiobesogenic potential. *Methods.* CWP (100 mg*·*kg*·*day) was administered in mice with diet-induced obesity for 8 weeks. The animals were divided into four groups: control normocaloric diet gavage with water (C) or coacervate (C-CWP), and high fat diet gavage with water (HF) or coacervate (HF-CWP). *Results.* HF-CWP reduced weight gain and serum lipid fractions and displayed reduced adiposity and insulin. Adiponectin was significantly higher in HF-CWP group when compared to the HF. The level of LPS in HF-W group was significantly higher when compared to HF-CWP. The IL-10 showed an inverse correlation between the levels of insulin and glucose in the mesenteric adipose tissue in the HF-CWP group. CWP promoted an increase in both phosphorylation AMPK and the amount of ATGL in the mesenteric adipose tissue in HF-CWP group. *Conclusion*. CWP was able to modulate effects, possibly due to its high biological value of proteins. We observed a protective effect against obesity and improved the inflammatory milieu of white adipose tissue.

## 1. Introduction

Over the past decades, the incidence of obesity in the population has increased severely, and it has become a public health challenge. Its etiology is multifactorial, encompassing environmental, dietary, physical inactivity, and genetic factors. Obesity is a complex disease associated with a high-calorie diet, which contributes to the development of several other chronic noncommunicable diseases [1]. Obesity is also associated with increased plasma endotoxin (lipopolysaccharide-LPS), saturated fatty acids [2, 3], and proinflammatory cytokines [3] all intricately involved in the development of comorbidities such as diabetes mellitus, hypertension, dyslipidemia, and metabolic syndrome.

The fat tissue is not merely an energy storage organ, as it plays crucial endocrine and immune roles. White adipose tissue (WAT) is an endocrine organ secreting pro- and anti-inflammatory adipokines such as tumor necrosis factor alpha (TNF-*α*) and interleukin-6 (IL-6), which are important inflammatory markers that stimulate the production of several proteins and proinflammatory cytokines in different cell types, via nuclear factor *κ*B activation (NF-*κ*B) [4]. In addition, endotoxin and saturated fatty acids act in the same pathway of NF-*κ*B activation. Many studies have shown that LPS (endotoxin) can activate these proteins in adipocytes, thereby increasing the gene expression of proinflammatory adipokines [5–7].

The main role of adipose lipolytic enzymes is to provide other tissues with FAs in case of energy demand. Triglyceride stored in the lipid droplet is first hydrolyzed by the adipose triglyceride lipase enzyme (ATGL), also known as desnutrin, releasing a diacylglycerol moiety and FA, which requires an abhydrolase domain containing 5 (ABHD-5) promoter to be activated. After hydrolysis by ATGL, diacylglycerols are then hydrolyzed sequentially by hormone-sensitive lipase (HSL) and monoglyceride lipase (MGL), producing nonesterified fatty acids (NEFAs) and glycerol [8]. Different lipases gain access to the lipid droplet when the proteins coating the vesicle (perilipins) are phosphorylated. Perilipin A normally prevents lipolysis of triglyceride by surrounding the lipid droplet, thus preventing the access of lipases.

Whey protein (WP) has been found to be an excellent prophylactic against obesity, because of the high biological value mediated by bioactive peptides. These act as antimicrobial agents, antihypertensive, and regulators of immune function, reducing body fat as well as a variety of related beneficial mechanisms for human health. They also have additional functions; for example, they have appetite suppressant effects [9], stimulate muscle protein synthesis, and regulate of body energy homeostasis [10]. There is plenty of evidence indicating the potential of the WP in anti-inflammatory and antioxidant effects of exercise [9, 11–13].

Chitosan complex coacervation with WP is composed of by-products from the processing of shrimp, crab (chitosan), and cheese, adding an environmental benefit to the product, as these by-products may be reused and not disposed of in landfill sites or released into rivers by producers [14]. In view of the above, together with the development of fractionation technique and whey protein preservation, employing the method may contribute to the recovery of this valuable nutrient and increase the expression of the functional properties [15]. Complex coacervation is defined as a colloidal separation forming two liquid phases. This process is essentially driven by attractive forces with opposite charges of a biopolymer. This phenomenon occurs by the formation of a system of balance between colloids and the diluted supernatant [16, 17]. Thus, the purpose of the present study was to investigate the prospect of a biotechnological process of complexation and separation of cheese whey proteins in chitosan and test their antiobesogenic potential through the modulation of inflammatory markers and lipolytic pathway present in obesity. 

## 2. Methods

### 2.1. Coacervation and Characterization

In this study we used sweet cheese whey (SW 1108 bag 25 kg) with 1.5% fat marketed by Company Alibra-PR. Dissolving 10 g of the whey powder in 100 mL of distilled water. Chitosan was used for the coacervation medium molar mass with 75–85% degree of deacetylation and viscosity of 200–800 cps (Sigma-Aldrich 44887-7). A concentration of 0.75 mg/mL of Chitosan was used. Chitosan was dissolved in citric acid (208 mmol/L) and, after this step, added to the cheese whey in a proportion of 1 : 1 under stirring at room temperature for 1 h. The pH of the solution of chitosan and WP was adjusted to 6 with NaOH (250 mmol/L) and solubilized at room temperature (±25°C) with stirring. Solids coagulated with chitosan known as coacervate (CWP) were collected by centrifugation (1300 g).

In order to obtain on average 30% of the protein coacervate 3 L cheese whey was used. Thus, samples of CWP were obtained for their chemical analysis of total lipids, total protein, and lactose. Finally, for measurements of samples of mineral micronutrients, Ca, K, Mg, and P, of cheese whey, CWP, and Chitosan, 100 *μ*g was subjected to an optical emission spectrometer for inductively coupled plasma (ICP OES, Perkin Elmer Optima 3000 DV, Norwalk, CT, USA). To determine the existing protein fractions in CWP the electrophoretic profile with reducing buffer containing 62.5 mM Tris-HCl, 20% glycerol, 2% sodium dodecyl sulfate(SDS) (10%), 5% *β*-mercaptoethanol, and bromophenol blue at pH 6.8 was performed.

### 2.2. Animals and Treatment

This study was approved by the Research Ethics Committee of the Universidade Federalde São Paulo, Escola Paulista de Medicina (UNIFESPEPM), as the search protocol number 0473/10. Experimental procedures are in accordance with Principles of Laboratory Animal Care formulated by the National Institutes of Health (National Institutes of Health Publication number 96-23, revised 1996).

Forty-nine male Swiss mice twelve-week-old from CEDEME (Centro de Desenvolvimento de Modelos Experimentais da Universidade Federal de São Paulo) were housed five in a cage in a standard experimental animal laboratory and kept under controlled conditions of light (12 h light-dark cycle with lights on at 6 am) and temperature (24 ± 1°C). All mice received water and food* ad libitum*. The animals were divided as follows: control diet plus tap water (C-W); control diet plus coacervate (C-CWP); high fat diet plus coacervate (HF-CWP); and high fat diet plus tap water (HF-W). All diets were prepared according to the recommendations of the American Institute of Nutrition [18] (Table 1). Coacervate (CWP) containing 100 mg*·*kg*·*day was given by gavage. The body weight gain was recorded twice a week.

### 2.3. Composition and Aspect of the Coacervate

**Figure 1:**
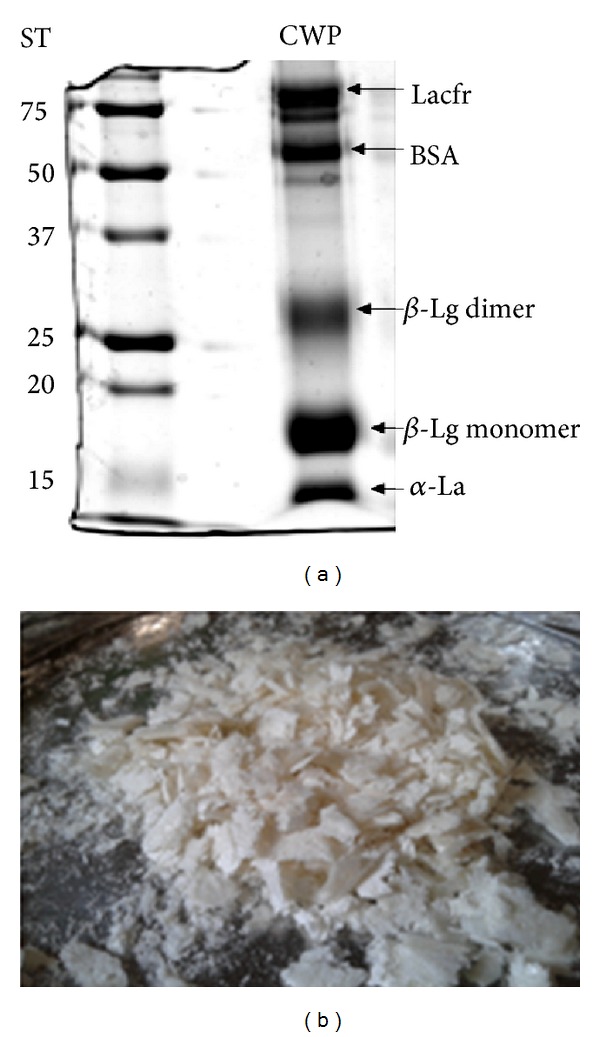
(a) Electrophoretic profile coacervate using 0.75 mg/ml of chitosan. ST: standard of different molecular weights, *α*-La: alpha-lactalbumin (14 kDa), *β*-Lg: beta-lactoglobulin monomer (18 kDa) and dimer (34 kDa), BSA: bovine albumin (66 kDa), and Lacfr: lactoferrin (86 kDa). (b) Aspect of CWP proteins in Chitosan, freeze-dried.

Figure 1(a) shows the protein profile of the coacervate by the presence of the major proteins of larger fractions, namely, alpha-lactalbumin (*α*-La-14 kDa), beta-lactoglobulin (*β*-Lg—18 kDa monomer and 34 kDa dimer form), bovine serum albumin (BSA—66 kD), and lactoferrin (Lacf—86 kDa). The CWP has a flocculation aspect (Figure 1(b)) consisting of proteins and micronutrients such as calcium, potassium, magnesium, phosphorus, and sodium (Table 2).

### 2.4. Fatty Acids Composition of Diets

For total lipid extraction, diet samples were homogenized in chloroform and methanol 2 : 1 (v/v), mixed, and incubated at room temperature for 5 min. Then, additional volumes of 1.25 mL chloroform and 1.25 mL deionized H_2_O were added, and finally, following being vigorously homogenized for 3 min, samples were centrifuged at 1000 rpm for 5 min at room temperature. The chloroform layer was dried under N_2_, and the total extract was converted into methyl esters and was analyzed in gas chromatography (GC), coupled with a flame ionizer detector (FID), (Varian GC 3900) and fatty acid profile was determined by calculating the retention time, using a pattern of fatty acids with known retention time (Supelco, 37 Components). The addition was initiated at a temperature of 170°C maintained for 1 minute and then a ramp of 2.5°C/min to a final temperature of 240°C, which was maintained for 5 minutes. The injector and detector were maintained at 250°C and 260°C, respectively. We used a column CP wax 52CB, with a thickness of 0.25 mm, internal diameter of 0.25 *μ*m, and length of 30 m,with hydrogen as the carrier gas at a linear velocity of 22 cm s^−1^.

### 2.5. Oral Glucose Tolerance Test (OGTT)

After 12 hours of fasting, blood was collected from the tail vein to assess basal glucose concentration. Then, a glucose (Merck) solution (1.4 g/kg) was administrated by gavage. Blood samples were collected after 15, 30, 45, 60, and 120 minutes to measure glucose concentration using a glucose analyzer (AccuCheck Roche).

### 2.6. Experimental Procedures

At the end of the experimental period, animals were fasted for 12 h overnight prior to being sacrificed by decapitation. Trunk blood was collected and immediately centrifuged (1125 g/15 min at 4°C). Serum was separated and stored at −80°C for later biochemical and hormonal determination. The adipose tissue depots, retroperitoneal (RET), mesenteric (MES), and epididymal (EPI), were dissected, weighed, immediately frozen in liquid nitrogen, and stored at −80°C.

### 2.7. Biochemical and Hormonal Serum Analyses

Serum concentrations of glucose, total cholesterol, triglycerides, and HDL-c were measured by an enzymatic colorimetric method using commercial kits (Labtest, Brazil). Concentrations of insulin and adiponectin were measured using specific enzyme-linked immunosorbent assay (ELISA) kits (Milipore and R&D Systems). LPS was determined using a commercial kit (Lonza).

### 2.8. Mesenteric Adipose Tissue TNF-*α*, IL-6, and IL-10 Protein Level Determined by ELISA

Following euthanasia, mesenteric adipose tissue was removed, homogenized into a specific total protein extraction buffer [1% Triton X-100, 100 mm Tris-HCl (pH 7.4), 100 mm sodium pyrophosphate, 100 mm sodium fluoride, 10 mm EDTA, 10 mm sodium orthovanadate, 2.0 mm phenylmethylsulfonyl fluoride, and 0.1 mg aprotinin/mL], and centrifuged at 12,000 g for 30 min at 4°C. The supernatant was saved, and the protein concentration was determined using the BCA assay (Bio-Rad, Hercules, California) with bovine serum albumin (BSA) as a reference. Quantitative assessment of TNF-*α*, IL-6, and IL-10 proteins was carried out by ELISA (DuoSet ELISA, R&D Systems, Minneapolis, MN) following the recommendations of the manufacturer. All samples were run as duplicates and the mean value was reported.

### 2.9. Protein Analysis by Western Blotting

After euthanasia, the mesenteric adipose tissue was dissected and homogenized in 1.0 mL of solubilization buffer at 4°C [1% Triton X-100, 100 mm Tris-HCl (pH 7.4), 100 mm sodium pyrophosphate, 100 mm sodium fluoride, 10 mm EDTA, 10 mm sodium orthovanadate, 2.0 mm phenylmethylsulfonyl fluoride, and 0.1 mg aprotinin/mL]. Insoluble material was removed by centrifugation for 30 min at 9000 g in a 70 Ti rotor (Beckman, Fullerton, CA, USA) at 4°C. The protein concentration of the supernatants was determined using the BCA assay (Bio-Rad, Hercules, CA, USA). Proteins were denatured by boiling (5 min) in a Laemmli sample buffer containing 100 mM DTT and were run on 10% sodium dodecyl sulfate polyacrylamide gel electrophoresis in a Bio-Rad miniature slab gel apparatus.

The proteins were electrotransferred from gels to nitrocellulose membranes for ~1.30 h/4 gels at 15 V (constant) in a Bio-Rad semidry transfer apparatus. Nonspecific protein binding to the nitrocellulose was reduced by preincubation for 2 h at 22°C in blocking buffer (1% BSA, 10 mM Tris, 150 mM NaCl, and 0.02% Tween 20). The nitrocellulose membranes were incubated overnight at 4°C with antibodies against hormone-sensitive lipase (HSL), adipose triglyceride lipase (ATGL), abhydrolase domain containing protein 5 (ABHD-5), perilipin A, phospho 5′ AMP-activated protein kinase (p-AMPK*α* 1 e 2 - Thr 172), and alpha-tubulin obtained from Santa Cruz Biotechnology (Santa Cruz, CA, USA) diluted 1 : 1000 with blocking buffer supplemented with 1% BSA and then washed for 30 min in blocking buffer without BSA. The blots were subsequently incubated with peroxidase-conjugated secondary antibody for 1 h at 22°C. To evaluate protein loading, the membranes were stripped and reblotted with an anti-alpha-tubulin antibody as appropriate. Specific bands were detected by chemiluminescence, and visualization/capture was performed by UVITEC gel-documentation system. Band intensities were quantified by optical densitometry of developed autoradiographs (Scion Image software, Scion Corporation, Frederick, MD, USA).

### 2.10. Statistical Analyses

All results are presented as mean ± standard error of the mean (SEM). Statistical significances were assessed using two-way analysis of variance (ANOVA) followed by Tukey's* post hoc* analysis to identify significant differences among the groups.* Pearson's* correlation was used to assess the associations between the analyzed variables. Differences were considered significant for (*P* ≤ 0.05) with the StatsDirect software.

## 3. Results

### 3.1. Body and Tissue Weights

**Figure 2:**
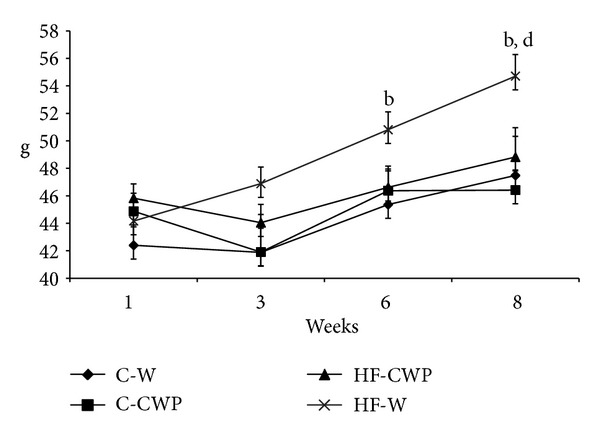
Evolution of the average gain in body mass (g) of mice for eight weeks of treatment with high fat diet (HF) or control normocaloric (C) associated with gavage of coacervate (CWP) or water (W). Data submitted with an average ± EPM. (b) C-W versus HF-W and (d) HF-CWP versus HF-W. (*P* < 0.05).

After six weeks of treatment, the hyperlipidic diet promoted an increase in the body weight when compared to the control (C-W versus HF-W). On the other hand, HF-CWP showed a lower body weight when compared to HF-W (Figure 2). The hyperlipid diet increased the relative mass of epididymal and mesenteric depot and adiposity (Σ of epididymal, retroperitoneal, and mesenteric relative weight) when compared to control group (C-W versus HF-W), while the association with coacervate reduced these parameters (HF-W versus HF-CWP). The retroperitoneal depot was increased in the HF-W group when compared to C-W one (Table 3).

### 3.2. Levels of Serum Lipids, Insulin, Adiponectin, Lipopolysaccharides, and OGTT

The hyperlipid diet increased the triacylglycerol (TAG) and VLDL when compared to control group (C-W versus HF-W), while the association with coacervate reduced these parameters (HF-W versus HF-CWP). Insulin level and HOMA index were increased in the animals fed with hyperlipidic diet (C-W versus HF-W). When associated with coacervate, the hyperlipid diet promoted an increase in the adiponectin and a decrease in LPS concentrations (HF-W versus HF-CWP) (Table 4).

**Figure 3:**
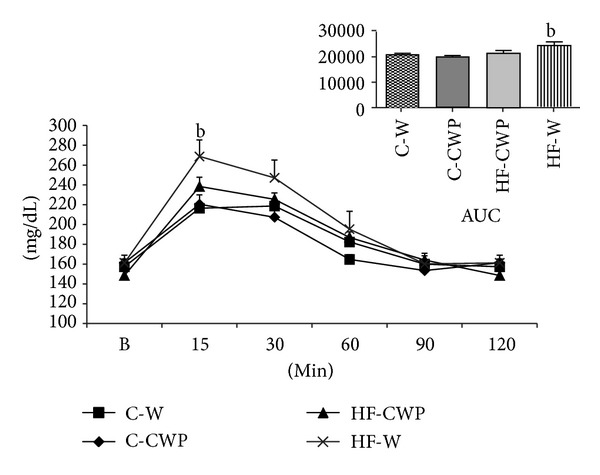
OGTT and AUC (area under the curve) after eight weeks of treatment with high fat diet (HF) or normocaloric control (C) associated with gavage of coacervate (CWP) or water (W). Glycemia in time zero (basal-B), 15, 30, 60, 90, and 120 minutes after gavage of 0.2 g/100 g body weight of glucose. Data submitted with an average ± EPM. (b) C-W versus HF-W. (*P* < 0.05).

The oral glucose tolerance test showed that the hyperlipidic diet promoted an increase at 15 minutes when compared to control (HF-W versus C-W). The AUC (area under the curve) analysis increased HF-W compared with C-W (Figure 3).

### 3.3. Concentration of IL-6, IL-10, and TNF-Alpha in the Mesenteric Adipose Tissue

There was a significant decrease in IL-10 concentrations in the animal fed with high fat diet when compared to animals fed the control diet (HF-W versus C-W). The concentration of IL-6 in C-CWP group was lower when compared to C-W group. The IL-10/TNF-*α* ratio in mesenteric tissue showed no significant differences (Table 5).

### 3.4. Expression of Proteins Involved in Lipolysis Pathway

**Figure 4:**
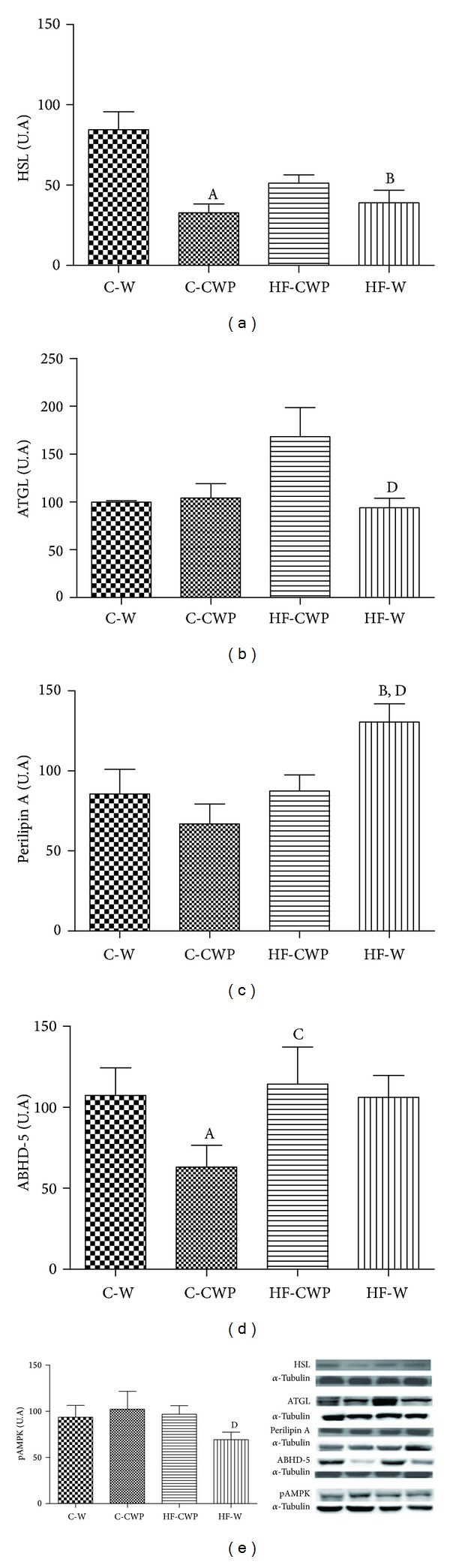
Protein expression of HSL (a); ATGL (b); perilipin A (c); ABHD-5 (d); and pAMPK (e) in the mesenteric adipose tissue. The data are expressed in arbitrary units (A.U). Data submitted with an average ± EPM. (A) C-W versus C-CWP; (B) C-W versus HF-W; (C) C-CWP versus HF-CWP; and (D) HF-CWP versus HF-W. (*P* < 0.05).

Figures 4(a), 4(b), 4(c), 4(d), and 4(e) show the data of protein expression of HSL, ATGL, Perilipin A, and ABHD-5 and AMPK activity, respectively, in mesenteric adipose tissue.

HSL protein expressions were reduced in C-CWP and HF-W when compared to the C-W group (Figure 4(a)). There was an increase in ATGL in HF-CWP group when compared to HF-W group (Figure 4(b)). Perilipin A was significantly higher in the HF-W group when compared to the C-W and HF-CWP groups (Figure 4(c)). There was a significant decrease in the protein expression of ABDH-5 in C-CWP when compared to C-W and HF-CWP groups (Figure 4(d)). The phosphorylation of AMPK (Figure 4(e)) was higher in HF-CWP compared to HF-W group.

### 3.5. Correlations

**Figure 5:**
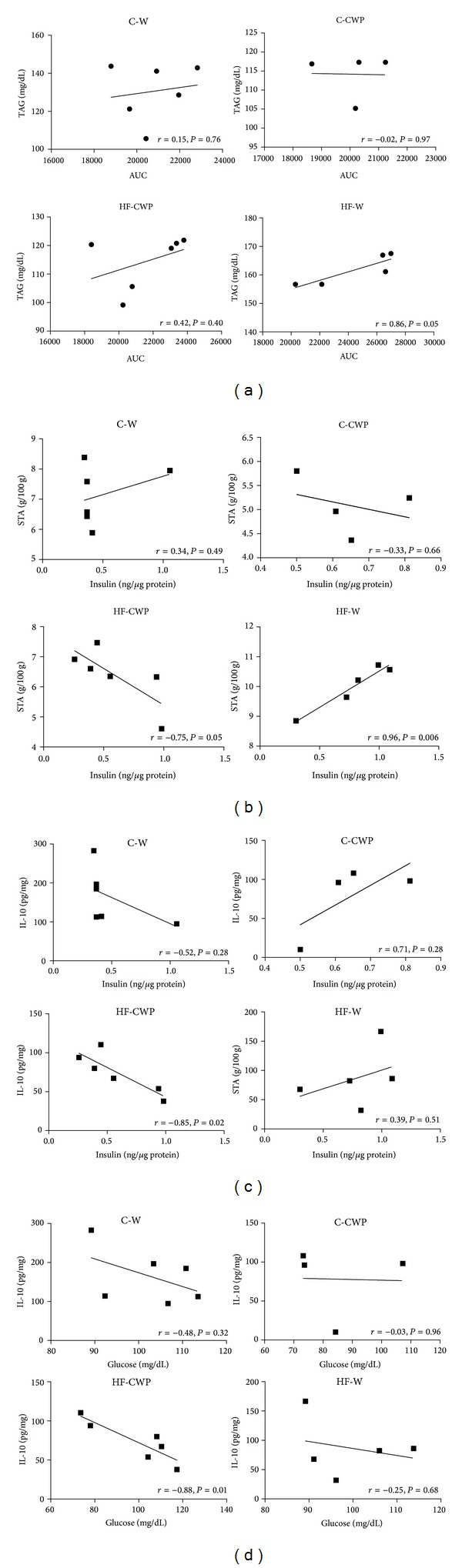
Correlation between different experimental groups: (a) TAG and AUC in different experimental groups. (b) Insulin and STA (sum of adipose tissues MES, RET, and EPI). (c) Insulin and IL-10 of serum and mesenteric adipose tissue, respectively, and (d) glucose and IL-10 of serum and mesenteric adipose tissue, respectively.

A positive correlation between AUC and TAG (*r* = 0.86  *P* < 0.05) was found in the HF-W group (Figure 5(a)).  Figure 5(b) shows a positive correlation between insulin levels and STA in HF-W (*r* = 0.96  *P* = 0.006) group. Figures 5(c) and 5(d) show an inverse correlation between insulin (*r* = −0.85  *P* = 0.02) (Figure 5(c)) and glucose (*r* = −0.88  *P* = 0.01) levels with IL-10 in the mesenteric adipose tissue in HF-CWP group (Figure 5(d)).

## 4. Discussion

Numerous procedures for isolation and recovery of WP have been investigated and reported [19]. The functional, physical, and chemical characteristics vary according to the procedures used to obtain these proteins. In our study, it was possible to recover an average OF 30% in WP (Table 2). If we compare these numbers with published data (which often use conventional techniques such as ultrafiltration (UF)), our method seems to be a low efficiency process [19]. However, cost-benefit considerations should be taken into account. For example, the UF method is expensive not only in terms of deployment but also in terms of operation. In addition to being cost-effective, the coacervation process promotes the separation of WP and obtains a low-calorie product.

The emergence of food compounds with health benefits may eventually become a good strategy to improve public health. In recent years, functional food has attracted the attention from scientific community, consumers, and food manufacturers. The list of nutraceuticals compounds (vitamins, probiotics, bioactive peptides, and antioxidants among others) is extensive, and scientific evidence seems to increasingly support the concept of health promotion through food ingredients [20, 21].

Functional foods are usually marketed as food containing ingredients technologically manipulated to perform a benefit for health [22]. Our study lends support to previous studies showing the effectiveness of CWP as a nutraceutical able to stabilize fat mass gain in animals fed with high fat diet. Our findings agree with the WP intake benefits extensively reported in literature [23–27]. As demonstrated, there was a decrease in body weight in the HF-CWP group when compared to the HF-W group, accompanied by a reduction in the adiposity.

It is now well established that excessive consumption of saturated fat is related to the development of dyslipidemias [28, 29], and this study further corroborates it, as the animals fed with high fat diet increased TAG and VLDL. Studies have demonstrated the insulinotropic effect of WP [9, 11, 30]. In this study, we did not find any significant difference in blood glucose between the groups assessed. Regarding insulin and HOMA index, the hyperlipidic diet showed significantly higher values. Our results demonstrated the effectiveness of this experimental model of obesity. However, CWP treatment promoted improved glucose and insulin tolerance. A study undertaken by Huang et al. [31] utilizing different protein sources (cheese whey, soy, red meat, and milk) in obese mice (induced by high fat diet) has found increased adiponectin concentration and reduced insulin when the animals were fed with whey protein cheese. In the present study, we also detected the critical role of CWP in modulating adiponectin, as the values were higher for the HF-CWP group when compared to HF-W group. These findings indicate that CWP may have functional effects. Yamauchi et al. [32] have demonstrated the potential of adiponectin in reducing insulin resistance by enhancing fatty acid oxidation, leading to a reduction in TAG content in obese diabetic rats.

There is strong evidence of an immunomodulatory role of WP [33]. Products containing WP may be beneficial in the treatment of certain diseases. Studies have shown that WP promotes improvement in the treatment of gastrointestinal symptoms of infant mice with rotavirus-induced diarrhea, a protective role in colorectal cancer in rats, reduced release of IL-6 in blood of rats undergoing transient ischemia/intestinal reperfusion and may provide protective effects against experimentally induced breast cancer in animals [25, 34, 35]. Most of the more recent studies in the literature, both* in vivo* and* in vitro*, have focused on the possible effects of these proteins in macrophages and lymphocytes. Although most proteins are degraded during the gastric digestion, certain cheese whey proteins, such as *β*-Lg, *α*-La, or GMP, are resistant to digestion and remain intact. Such proteins can directly stimulate leukocyte after their digestive absorption. Thus, it is important to understand the mechanisms underlying the impact of these proteins on immunity, stimulating anti-inflammatory processes in the body [33].

The IL-10 is a pleiotropic cytokine that controls inflammatory processes by eliminating the proinflammatory cytokines production such as IL-1, IL-6, and IL-8, and TNF-*α* is produced mainly by monocytes, macrophages, lymphocytes, mast cells, and mature adipocytes [14, 36]. The IL-10/TNF-*α* ratio has been considered an important indicator of inflammatory status as low values are often associated with increased morbidity and mortality risk. We did not observe an increase of the IL-10/TNF-*α* ratio in MES of group HF-CWP. We believe that because of the short period of the treatment the animals may not have developed their proinflammatory state. With increased length of treatment, we believe find results more expressive. Another possibility is that the coacervate may have protected the mice from a proinflammatory state triggered by the treatment diet, leading to the counterbalance of IL-10 unnecessary in HF-CWP group. However, a study involving a longer treatment period may be required to discern this possible effect.

In this sense, there is an immunomodulatory mechanism underlying CWP, most likely the IL-10 cytokine, which has a homeostatic metabolic effect in the mesenteric adipose tissue. Our result suggests that IL-10 may be a positive regulator of insulin sensitivity and increased glucose uptake. This mechanism can protect the adipose tissue against insulin resistance. Although the precise origin of the unchecked inflammatory response in obesity is still unclear, it is well known that in obesity the overproduction of proinflammatory cytokines affects metabolism. For example, TNF-*α* contributes to the inability of cells to respond to insulin and to increased levels of insulin [37], and IL-10 was associated with other variables closely linked to insulin sensitivity, such as fasting and postload insulin concentrations, HDL cholesterol, and triglyceride levels. Besides the tissue-specific effect of CWP, we showed a systemic effect in the decrease in the LPS serum level.

Regarding the composition of the CWP, we may highlight the presence of *α*-La and *β*-Lg protein, the major protein present in the coacervate, which has been proven effective in suppressing the release of proinflammatory cytokines [27, 38]. The presence of these proteins is a great indication that the coacervate components are able to modulate the proinflammatory milieu promoted by hyperlipidic diet. In another study by our group, mice, previously treated with high fat diet and fed with a supplementation of CWP (gavage, 36 mg protein/kg of body weight), showed a positive correlation between IL-10 and TNF-alpha in mesenteric adipose tissue, retroperitoneal adipose tissue, and liver tissue. We also observed a positive correlation between lipopolysaccharide and IL-10 in the liver tissue. Therefore, pretreatment with high fat diet promoted metabolic alterations and inflammation, and CWP modulated the inflammatory milieu [14].

Evidence suggests that eating WP causes the decrease in calorie intake, increased basal energy expenditure, and modulates insulin sensitivity and glucose homeostasis, leading to changes in lipid metabolism in adipose tissue, liver, and muscle [39–42]. The AMPK and adiponectin are key molecules to metabolic responses in different tissues [43, 44]; they are involved in the preventive response against negative physiological processes caused by the consumption of a diet high in saturated fatty acids. The activation of AMPK by bioactive components of foods or medicines has been regarded as goal, since it may reverse the metabolic changes associated with obesity and type 2 diabetes [45]. It is also known that adiponectin can activate AMPK in white adipose tissue [46]. The animals treated with coacervate showed an increase in AMPK activation associated with the decrease in HSL and increase in ATGL protein expression in mesenteric adipose tissue. Similar results were reported in the review undertaken by Bijland et al. [47].

A study conducted by Gaidhu et al. [43] showed that AMPK activation stimulated by AICAR (*5-aminoimidazol*-*4*-*carboxamida ribonucleotídeo)* initially promoted inhibition of lipolysis in adipocytes isolated as* in vivo*, reflecting a decrease in free fatty acid in serum. On the other hand, prolonged treatment with AICAR promoted an increase in lipolysis, which the authors attributed to an increase in the content of ATGL and reduced activity of HSL [48]. However, clear-cut conclusions are difficult to arrive at, due to a lack of tools for manipulating assays using specific AMPK. Furthermore, the overall effect of AMPK activation of lipolysis is still controversial. The duration and mode of activation of AMPK may be of particular importance when it comes to a process aimed at reducing the proinflammatory state caused by increased lipolysis [47]. In addition, adiponectin can suppress the activation of HSL, without changing ATGL and ABHD-5 in adipocytes in order to modulate a homeostatic control of lipolysis to avoid lipotoxicity [49].

Lipolysis does seem to play a crucial physiological role by recruiting a source of energy mobilized in times of stress and/or energy deprivation. Moreover, the very significant reduction in lipolysis is clearly harmful, as demonstrated in the clinical domain by the syndromes resulting from deficiencies in the lipolytic apparatus [50]. Given that, it is reasonable to question whether the inhibition of lipolysis, via HSL induced by CWP, is helpful or harmful, since enhanced lipolytic activity and concomitant increase of free fatty acid in the circulation is clearly deleterious and leads to several comorbidities. To clearly determine this, however, an analysis of free fatty acid and endogenous glycerol concentrations would need to be carried out.

Another interesting finding was the significantly higher protein expression of perilipin A (52%) in HF-W group, which also refers to larger deposits of triglycerides, since this protein is primarily anchored around the droplets of neutral lipids in adipocytes. This is in line with studies showing that increased protein expression perilipin A leads to increased storage of triglycerides by reducing its hydrolytic rate. [51].

Finally, there is plenty of evidence suggesting that the intake of WP may lower consumption of calories, increase baseline energy expenditure, and improve insulin sensitivity and glucose homeostasis, thus leading to changes in lipid metabolism in adipose tissue, liver, and muscle [39–42].

## 5. Conclusion

CWP were able to promote nutritional and physiological improvements in HF-CWP group, such as reduction in body mass and decreased serum lipid levels followed by decreased serum insulin and LPS. In addition, intervention with CWP resulted in higher adiponectin contents and attenuated processes that would lead to glucose intolerance. Therefore, CWP could play a beneficial role, in some way, in modulating lipolysis in animals treated with hyperlipidic diet.

## Figures and Tables

**Table 1:** Composition of the control diet and diet enriched with saturated fatty acids according to AIN-93. Coacervate was resuspended in 300 *µ*L of water.

Components (%)	Control diet (C)	High fat diet (HF)
Corn starch	72.07	40.87
Casein	14.0	14.0
Soybean oil	4.0	4.0
Lard	—	31.2
Cellulose	5.0	5.0
Vitamin mix	1.0	1.0
Mineral mix	3.5	3.5
L-cystine	0.18	0.18
Choline bitartrate	0.25	0.25
Butyl hydroquinone. g/kg	0.008	0.008
Energy. kcal/kg	3,802.8	5,362.8
Treatment by gavage		
Coacervate (CWP)	(C-CWP) 100 mg*·*kg*·*day	(HF-CWP) 100 mg*·*kg*·*day
Water (W)	(C-W) 300 *µ*L*·*day	(HF-W) 300 *µ*L*·*day
Fatty acids (%)		
Saturated (SFA)	17.12	34.13
Monounsaturated (MUFA)	25.63	39.14
Polyunsaturated (PUFA)	57.25	26.67
PUFA n3	4.32	6.37
PUFA n6	52.65	19.98

**Table 2:** Partial composition of micronutrients by ICP and chemical macronutrients of CWP, WP, and Chitosan.

Nutritional composition			
g/100 g			
	CWP	WP	Chitosan
Proteins	30	35	—
Lactose	1.7	40	—
Lipids	0.4	1.5	—
Ca	0.47	0.55	0.07
K	0.37	1.69	0.010
Mg	0.06	0.11	<0.001
P	0.46	0.53	0.003
Na	0.81	1.32	0.26

**Table 3:** Total mass (g), delta on the body mass gain (g/100 g body weight), and relative mass of tissue (RMT) (g/100 g body weight) of mice treated with high fat diet (HF) or control normocaloric (C) associated with gavage of coacervate (CWP) or water (W).

	C-W (*n* = 10)	C-CWP (*n* = 10)	HF-CWP (*n* = 19)	HF-W (*n* = 19)
RTM (g/100 g)				
EPI	4.04 ± 0.17	3.52 ± 0.21	4.06 ± 0.28	4.93 ± 0.44^b,d^
MES	1.80 ± 0.24	1.29 ± 0.21	1.72 ± 0.1	2.6 ± 0.36^b,d^
RET	1.12 ± 0.10	0.82 ± 0.04	1.35 ± 0.12	1.48 ± 0.18^b^
Σ adipose tissue	6.97 ± 0.38	5.64 ± 0.41	7.14 ± 0.46	9.02 ± 0.55^b,d^

EPI: epididymal; MES: mesenteric; RET: retroperitoneal. Data submitted with an average ± EPM. ^b^C-W versus HF-W and ^d^HF-CWP versus HF-W. (*P* < 0.05).

**Table 4:** Serum of triacylglycerol, total cholesterol, glucose, insulin, and adiponectin of mice treated with high fat diet (HF) or control normocaloric (C) associated with gavage of coacervate (CWP) or water (W).

Serum measurements	C-W (*n* = 10)	C-CWP (*n* = 11)	HF-CWP (*n* = 17)	HF-W (*n* = 17)
Triacylglycerol (mg/dL)	133.37 ± 4.28	127.09 ± 4.85	129.30 ± 4.31	141.55 ± 3.94^b,d^
Total cholesterol (mg/dL)	128.75 ± 4.22	129.46 ± 4.76	128.99 ± 3.35	150.03 ± 6.00
LDL (mg/dL)	68.81 ± 1.75	73.18 ± 3.07	75.66 ± 1.64	80.3 ± 3.28
VLDL (mg/dL)	26.32 ± 0.86	25.42 ± 0.97	25.86 ± 0.86	28.92 ± 0.76^b,d^
HDL (mg/dL)	33.62 ± 1.75	36.71 ± 3.07	35.13 ± 1.64	40.83 ± 3.28
Glucose (mg/dL)	100.27 ± 2.69	89.84 ± 4.45	100.80 ± 3.58	105.28 ± 3.47
Insulin (ng/mL)	0.51 ± 0.03	0.64 ± 0.01	0.65 ± 0.01	0.75 ± 0.02^b^
HOMA-IR	0.89 ± 0.26	0.97 ± 0.15	1.12 ± 0.21	1.25 ± 0.19^b^
Adiponectin (ng/mL)	3.55 ± 0.47	3.98 ± 0.26	4.04 ± 0.10	3.34 ± 0.29^d^
Lipopolysaccharides (EU/mL)	2.58 ± 0.98	1.61 ± 0.66	1.49 ± 0.50	3.07 ± 0.97^d^

Data submitted with an average ± EPM. ^b^C-W versus HF-W and ^d^HF-CWP versus HF-W. (*P* < 0.05).

**Table 5:** Concentrations of IL-6, TNF-*α*, IL-10, and IL-10/TNF-*α* (pg/mg of total protein content) in different experimental groups.

	C-W (*n* = 6)	C-CWP (*n* = 7)	HF-CWP (*n* = 12)	HF-W (*n* = 12)
IL-6	329.69 ± 43.64	132.08 ± 24.10^a^	215.80 ± 49.70	241.61 ± 29.27
TNF-α	111.93 ± 21.71	92.60 ± 22.86	54.48 ± 8.27	69.38 ± 17.35
IL-10	153.36 ± 27.01	119.30 ± 29.04	65.05 ± 9.96	100.74 ± 20.97^b^
IL-10/TNF-*α*	1.37 ± 0.027	1.28 ± 0.28	1.19 ± 0.10	1.45 ± 0.67

Data submitted with an average ± EPM. ^a^C-W versus C-CWP; ^b^C-W versus HF-W. (*P* < 0.05).

## Abbreviations

ABHD-5: Abhydrolase domain containing 5
AUC: Area under the curve
CWP: Coacervate
C-CWP: Control diet plus coacervate
C-W: Control diet plus tap water
HF-CWP: High fat diet plus coacervate
HF-W: High fat diet plus tap water
HSL: Hormone-sensitive lipase
LPS: Lipopolysaccharide
MGL: Monoglyceride lipase
NEFAs: Nonesterified fatty acid
p-AMPK*α* 1 e 2 - Thr 172: Phospho 5′ AMP-activated protein kinase
TAG: Triacylglycerol
OGTT: Oral glucose tolerance test
ATGL: Triglyceride lipase enzyme
WP: Whey protein.

## References

[1] Gregor MF, Hotamisligil GS (2011). Inflammatory mechanisms in obesity. *Annual Review of Immunology*.

[2] Kueht ML, McFarlin BK, Lee RE (2009). Severely obese have greater LPS-stimulated TNF-α production than normal weight African-American women. *Obesity*.

[3] Eilers PHC, Westerterp-plantenga MS, Kooistra T, Biology H, Maastricht MSW (2004). Leptin and the proinflammatory state associated with human obesity. *Journal of Clinical Endocrinology & Metabolism*.

[4] Fonseca-Alaniz MH, Takada J, Alonso-Vale MIC, Lima FB (2007). Adipose tissue as an endocrine organ: from theory to practice. *Jornal de Pediatria*.

[5] Song MJ, Kim KH, Yoon JM, Kim JB (2006). Activation of Toll-like receptor 4 is associated with insulin resistance in adipocytes. *Biochemical and Biophysical Research Communications*.

[6] Chait A, Kim F (2010). Saturated fatty acids and inflammation: who pays the toll?. *Arteriosclerosis, Thrombosis, and Vascular Biology*.

[7] Park J, Sung SC, Choi AH (2006). Increase in glucose-6-phosphate dehydrogenase in adipocytes stimulates oxidative stress and inflammatory signals. *Diabetes*.

[8] Vázquez-Vela MEF, Torres N, Tovar AR (2008). White adipose tissue as endocrine organ and its role in obesity. *Archives of Medical Research*.

[9] Hall WL, Millward DJ, Long SJ, Morgan LM (2003). Casein and whey exert different effects on plasma amino acid profiles, gastrointestinal hormone secretion and appetite. *British Journal of Nutrition*.

[10] Anthony TG, McDaniel BJ, Knoll P, Bunpo P, Paul GL, McNurlan MA (2007). Feeding meals containing soy or whey protein after exercise stimulates protein synthesis and translation initiation in the skeletal muscle of male rats. *Journal of Nutrition*.

[11] Shi J, Tauriainen E, Martonen E (2011). Whey protein isolate protects against diet-induced obesity and fatty liver formation. *International Dairy Journal*.

[12] Rusu D, Drouin R, Pouliot Y, Gauthier S, Poubelle PE (2009). A bovine whey protein extract can enhance innate immunity by priming normal human blood neutrophils. *Journal of Nutrition*.

[13] Tranberg B, Hellgren LI, Lykkesfeldt J (2013). Whey protein reduces early life weight gain in mice fed a high-fat diet. *PLoS One*.

[14] Moreno MF, de Morais Honorato de Souza GI, Hachul ACL (2014). Coacervate whey protein improves inflammatory milieu in mice fed with high-fat diet. *Nutrition & Metabolism*.

[15] Hidalgo J, Hansen PMT (1970). *Selective Precipitation of Whey Proteins with Carboxymethylcellulose*.

[16] Tolstoguzov VB (1991). Functional properties of food proteins and role of protein-polysaccharide interaction. *Food Hydrocolloids*.

[17] Cooper CL, Dubin PL, Kayitmazer AB, Turksen S (2005). Polyelectrolyte-protein complexes. *Current Opinion in Colloid and Interface Science*.

[18] Reeves PG, Nielsen FH, Fahey GC (1993). AIN-93 purified diets for laboratory rodents: final report of the American Institute of Nutrition ad hoc writing committee on the reformulation of the AIN-76A rodent diet. *Journal of Nutrition*.

[19] de Wit JN (2001). *Lecturer's Handbook on Whey and Whey Products*.

[20] Chen L, Remondetto GE, Subirade M (2006). Food protein-based materials as nutraceutical delivery systems. *Trends in Food Science and Technology*.

[21] Caillard R, Guillet-Nicolas R, Kleitz F, Subirade M (2012). Tabletability of whey protein isolates. *International Dairy Journal*.

[22] Niva M (2007). “All foods affect health”: understandings of functional foods and healthy eating among health-oriented Finns. *Appetite*.

[23] Bounous G, Gold P (1991). The biological activity of undenatured dietary whey proteins: role of glutathione. *Clinical and Investigative Medicine*.

[24] Iskandar MM, Dauletbaev N, Kubow S, Mawji N, Lands LC (2013). Whey protein hydrolysates decrease IL-8 secretion in lipopolysaccharide (LPS)-stimulated respiratory epithelial cells by affecting LPS binding to Toll-like receptor 4. *British Journal of Nutrition*.

[25] Hakkak R, Korourian S, Ronis MJJ, Johnston JM, Badger TM (2001). Dietary whey protein protects against azoxymethane-induced colon tumors in male rats. *Cancer Epidemiology Biomarkers and Prevention*.

[26] Rosaneli CF, Bighetti AE, Antonio MA, Carvalho JE, Sgarbieri VC (2002). Efficacy of a whey protein concentrate on the inhibition of stomach ulcerative lesions caused by ethanol ingestion. *Journal of Medicinal Food*.

[27] Pal S, Ellis V, Dhaliwal S (2010). Effects of whey protein isolate on body composition, lipids, insulin and glucose in overweight and obese individuals. *British Journal of Nutrition*.

[28] Zhao S, Wang J, Song X, Zhang X, Ge C, Gao S (2010). Impact of dietary protein on lipid metabolism-related gene expression in porcine adipose tissue. *Nutrition and Metabolism*.

[29] Cintra DEC, Costa AV, Peluzio MDCG, Matta SLP, Silva MTC, Costa NMB (2006). Lipid profile of rats fed high-fat diets based on flaxseed, peanut, trout, or chicken skin. *Nutrition*.

[30] Frid AH, Nilsson M, Holst JJ, Björck IME (2005). Effect of whey on blood glucose and insulin responses to composite breakfast and lunch meals in type 2 diabetic subjects. *The American Journal of Clinical Nutrition*.

[31] Huang X-F, Liu Y, Rahardjo GL, Mclennan PL, Tapsell LC, Buttemer WA (2008). Effects of diets high in whey, soy, red meat and milk protein on body weight maintenance in diet-induced obesity in mice. *Nutrition and Dietetics*.

[32] Yamauchi T, Kamon J, Waki H (2001). The fat-derived hormone adiponectin reverses insulin resistance associated with both lipoatrophy and obesity. *Nature Medicine*.

[33] Rusu D, Drouin R, Pouliot Y, Gauthier S, Poubelle PE (2010). A bovine whey protein extract stimulates human neutrophils to generate bioactive IL-1Ra through a NF-*κ*B- and MAPK-dependent mechanism. *Journal of Nutrition*.

[34] Wolber FM, Broomfield AM, Fray L, Cross ML, Dey D (2005). Supplemental dietary whey protein concentrate reduces rotavirus-induced disease symptoms in suckling mice. *Journal of Nutrition*.

[35] Yamaguchi M, Uchida M (2007). α-lactalbumin suppresses interleukin-6 release after intestinal ischemia/reperfusion via nitric oxide in rats. *Inflammopharmacology*.

[36] Juge-Aubry CE, Somm E, Pernin A (2005). Adipose tissue is a regulated source of interleukin-10. *Cytokine*.

[37] Han JM, Patterson SJ, Speck M, Ehses JA, Levings MK (2014). Insulin inhibits IL-10-mediated regulatory T cell function: implications for obesity. *The Journal of Immunology*.

[38] Yamaguchi M, Uchida M (2007). α-Lactalbumin suppresses interleukin-6 release after intestinal ischemia/reperfusion via nitric oxide in rats. *Inflammopharmacology*.

[39] Zemel MB (2004). Role of calcium and dairy products in energy partitioning and weight management. *The American Journal of Clinical Nutrition*.

[40] Lönnerdal B (2003). Nutritional and physiologic significance of human milk proteins. *The American Journal of Clinical Nutrition*.

[41] Bouthegourd J-CJ, Roseau SM, Makarios-Lahham L, Leruyet PM, Tomé DG, Even PC (2002). A preexercise α-lactalbumin-enriched whey protein meal preserves lipid oxidation and decreases adiposity in rats. *American Journal of Physiology: Endocrinology and Metabolism*.

[42] McAllan L, Cotter PD, Roche HM, Korpela R, Nilaweera KN (2012). Bioactivity in whey proteins influencing energy balance. *Journal of Metabolic Syndrome*.

[43] Gaidhu MP, Anthony NM, Patel P, Hawke TJ, Ceddia RB (2010). Dysregulation of lipolysis and lipid metabolism in visceral and subcutaneous adipocytes by high-fat diet: Role of ATGL, HSL, and AMPK. *The American Journal of Physiology—Cell Physiology*.

[44] Kadowaki T, Yamauchi T (2005). Adiponectin and adiponectin receptors. *Endocrine Reviews*.

[45] Zhang BB, Zhou G, Li C (2009). AMPK: an emerging drug target for diabetes and the metabolic syndrome. *Cell Metabolism*.

[46] Huypens P, Quartier E, Pipeleers D, van de Casteele M (2005). Metformin reduces adiponectin protein expression and release in 3T3-L1 adipocytes involving activation of AMP activated protein kinase. *European Journal of Pharmacology*.

[47] Bijland S, Mancini SJ, Salt IP (2013). Role of AMP-activated protein kinase in adipose tissue metabolism and inflammation. *Clinical Science*.

[48] Gaidhu MP, Fediuc S, Anthony NM (2009). Prolonged AICAR-induced AMP-kinase activation promotes energy dissipation in white adipocytes: novel mechanisms integrating HSL and ATGL. *Journal of Lipid Research*.

[49] Qiao L, Kinney B, Schaack J, Shao J (2011). Adiponectin inhibits lipolysis in mouse adipocytes. *Diabetes*.

[50] Santamarina-Fojo S (1998). The familial chylomicronemia syndrome. *Endocrinology and Metabolism Clinics of North America*.

[51] Brasaemle DL, Rubin B, Harten IA, Gruia-Gray J, Kimmel AR, Londos C (2000). Perilipin A increases triacylglycerol storage by decreasing the rate of triacylglycerol hydrolysis. *The Journal of Biological Chemistry*.

